# Effects of Different Concentrations of Carbamide Peroxide on Color, Surface Roughness, and Hardness of CAD/CAM Dental Ceramics

**DOI:** 10.1002/cre2.916

**Published:** 2024-07-05

**Authors:** Saman Jamshidi, Homayoun Alaghemand, Behnaz Esmaeili, Hemmat Gholinia

**Affiliations:** ^1^ Department of Restorative Dentistry, School of Dentistry Babol University of Medical Sciences Babol Iran; ^2^ Department of Restorative Dentistry, Dental Materials Research Center, School of Dentistry, Health Research Institute Babol University of Medical Sciences Babol Iran; ^3^ Health Research Department, Oral Health Research Center, Health Research Institute Babol University of Medical Sciences Babol Iran; ^4^ Health Research Department, School of Dentistry, Health Research Institute Babol University of Medical Sciences Babol Iran

**Keywords:** carbamide peroxide, ceramics, color, hardness

## Abstract

**Objectives:**

This study assessed the effects of 15% and 20% carbamide peroxide (CP) on color, surface roughness, and hardness of computer‐aided design/computer‐aided manufacturing (CAD/CAM) dental ceramics.

**Materials and Methods:**

This in vitro study was conducted on 120 Vita Mark II, Celtra Duo, and Suprinity CAD/CAM ceramic specimens. The ceramic specimens in each group (*n* = 40) were randomly assigned to two subgroups (*n* = 20) for polishing and glazing, and their baseline color, surface roughness (Ra), and hardness were assessed. In each subgroup, half of the specimens were exposed to 15% CP, while the other half were exposed to 20% CP. Their color change (Δ*E*), surface roughness, and hardness were then measured again. Surface roughness, hardness, and color were analyzed sequentially by profilometer, Vickers hardness tester, and spectrophotometer, respectively. Data were analyzed by repeated measures ANOVA, one‐way ANOVA, and post hoc Bonferroni test (*α* = 0.05).

**Results:**

The surface roughness of all groups significantly increased after bleaching treatment (*p* < 0.05). Surface hardness of all groups decreased after bleaching treatment, but this reduction was only significant in Vita Mark II subgroups (glazed, polished, 15%, and 20% CP). The Δ*E* was not clinically and visually perceivable in any group.

**Conclusion:**

The present results revealed that concentration of CP and type of surface treatment affected the surface properties of CAD/CAM ceramics. Type of surface treatment only affected the surface hardness of Vita Mark II ceramics (*p* < 0.05). Concentration of CP had a significant effect only on polished Vita Mark II.

## 1 Introduction

The esthetic appearance of a smile depends on the color, shape, and leveling and alignment of teeth (Tavangar, Mousavipour, and Ansarifard 2021). Several cosmetic procedures such as tooth bleaching, microabrasion, macroabrasion, laminate veneers, and ceramic restorations are commonly used for improvement of smile attractiveness by changing the color, form, texture, or position of teeth (AlQahtani 2013).

Since the advent of computer‐aided design/computer‐aided manufacturing (CAD/CAM) technology, all‐ceramic restorations are increasingly used for restorative and cosmetic dental procedures (Della Bona, Nogueira, and Pecho 2014). Excellent mechanical properties such as optimal mechanical resistance, and favorable translucency have contributed to the popularity of all‐ceramic CAD/CAM restorations (Kim and Kim 2014). However, despite the common application of ceramics for dental restorations, they may experience surface changes due to pH alterations in the oral cavity. Chemical softening of the restorative materials caused by bleaching agents may affect their microhardness and surface roughness and, therefore, the clinical longevity of tooth‐colored restorations (Demir, Karci, and Ozcan 2020).

Dental bleaching is a minimally invasive cosmetic dental procedure with optimal esthetic results, which is highly popular for whitening of tooth shade due to its conservative nature (AlQahtani 2013; Malkondu et al. 2011).

However, it may be associated with some risks since it is not often performed under the supervision of professionals (Demir, Karci, and Ozcan 2020); Zavanelli et al. 2011).

The side effects of tooth bleaching have been the topic of many investigations (Albanai, Gillam, and Taylor 2015; Barbosa et al. 2024; de Arruda et al. 2012). Sensitivity, mucosal irritation, and alteration of enamel surface are some of the reported hazardous effects of bleaching on natural teeth (Tinastepe et al. 2021), and it has been confirmed that bleaching agents can adversely affect dental restorative materials such as dental amalgam, alloys, glass ionomer cements, and composite resins (Yu et al. 2015). Bleaching agents also have destructive effects on dental ceramics and can adversely affect their surface roughness (Abu‐Eittah and Mandour 2011; El‐Murr, Ruel, and St‐Georges 2011; Malkondu et al. 2011) and microhardness (El‐Murr, Ruel, and St‐Georges 2011; Alamoush et al. 2023).

Despite the extensive use of dental bleaching, literature is conflicting regarding its effects on properties of restorative materials. Also, the effects of bleaching agents on physical and chemical properties of dental ceramics have yet to be fully elucidated (El‐Murr, Ruel, and St‐Georges 2011). In particular, controversy exists regarding the effects of bleaching agents on roughness, color, and hardness of different dental ceramics (El‐Murr, Ruel, and St‐Georges 2011). Therefore, we decided to assess the effects of 15% and 20% carbamide peroxide (CP), which are commonly used for home bleaching, on color, surface roughness, and hardness of glazed and polished CAD/CAM dental ceramics. The null hypothesis of this study was that home bleaching with CP 15% and 20% has no effect on surface hardness, roughness, and color of polished and glazed Vita Mark II, Celtra Duo, and Suprinity.

## 2 Materials and Methods

This in vitro study was conducted on 120 Vita Mark II, Celtra Duo, and Suprinity CAD/CAM dental ceramic specimens with A3 shade. Ethics committee of Babol University of Medical Sciences approved the study protocol (IR.MUBABOL.HRI.REC.1401.088).

A total number of 120 ceramic samples, including 40 of each of Celta Duo, Suprinity, and Vita Mark II ceramics, were selected and each group was divided into two categories: polish and glaze (*n* = 20), and then half of them were treated with CP 15% and half others were treated with CP 20% (*n* = 10). The specimens were numbered and randomization was performed blindly using the numbers by a colleague of the Dental Materials Research Center.

**Figure 1:**
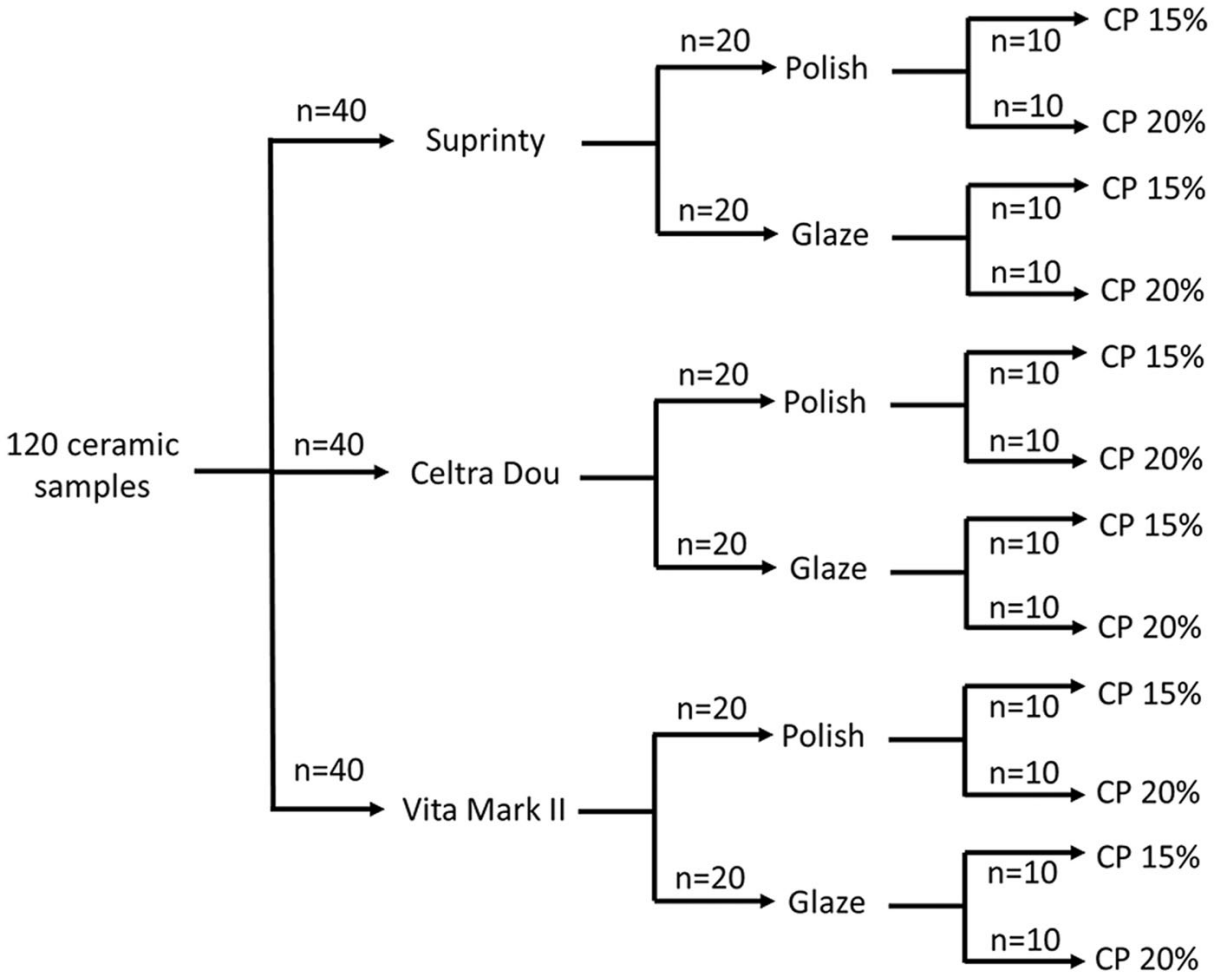
Classification of samples.

The number of samples was considered to be 10 specimens in each subgroup according to the study by Ünver and Yildirim (2022) (Figure 1).

### 2.1 Specimen Preparation

Ceramic specimens were milled from CAD/CAM ceramic blocks of Vita Mark II feldspathic porcelain (VITA Zahnfabrik, Bad Säckingen, Germany), and Suprinity (VITA Suprinity, Zahnfabrik, Bad Säckingen, Germany) and Celtra Duo (Dentsply DeTrey GmbH, Konstanz, Germany) high‐translucency zirconia‐reinforced lithium silicate ceramics with 1‐mm thickness (for the purpose of standardization) using a milling machine (Delta precision section machine, Mashhad, Iran). Forty specimens measuring 12 × 14 × 1 mm were fabricated from each ceramic type (Ünver and Yildirim 2022). Each specimen was fabricated from one ceramic block. All the ceramics used in this study were A3 shade.

For the purpose of standardization, the specimen surfaces were finished with a fine‐grit fissure bur (D + Z Diamant GmbH, Kalletal, Germany) and polished with 600‐grit silicon carbide abrasive paper (Daryakenari, Alaghehmand, and Bijani 2019). The ceramic specimens in each group were then randomly assigned to two subgroups for glazing and polishing (*n* = 20 in each subgroup).

### 2.2 Glazing

#### 2.2.1 Vita Mark II Specimens

Vita furnace (Certified VITA furnaces, Bad Säckingen, Germany) was used for this purpose as per the manufacturer's instructions. The glaze material (VITA Glaze LT; VITA Zahnfabrik, Bad Säckingen, Germany) was applied on Vitablock Mark II feldspathic porcelain specimens and then they were heated at 600°C for 4 min. The temperature was then gradually increased at a rate of 75°C/min to reach 900°C. The specimens remained at this temperature for 1 min. The temperature range was 600°C–900°C and the glazing time was 9 min. Glazing was accomplished as such.

#### 2.2.2 Celtra Duo Specimens

The glaze material (Dentsply Sirona Universal Spray Glaze; DeguDent GmbH, Hanau, Germany) was applied on Celtra Duo zirconia‐reinforced lithium silicate specimens and they were heated in a furnace at 480°C for 4 min. Next, the temperature was increased at a rate of 60°C/min within 5 min and 40 s under vacuum until the furnace temperature reached 820°C. The specimens remained at this temperature for 1 min to allow completion of crystallization and glazing (480°C–820°C, 10 min and 40 s).

#### 2.2.3 Suprinity Specimens

Suprinity Specimens: The glaze material (VITA AKZENT Plus, Bad Säckingen, Germany) was applied on Suprinity ceramic specimens and they were heated at 380°C for 4 min in a furnace. Next, the temperature increased at a rate of 55°C/min within 8 min and 21 s under vacuum to reach 840°C. The specimens remained at this temperature for 8 min to accomplish crystallization and glazing (380°C–840°C, 1 min and 22 s).

### 2.3 Polishing

A polishing kit (DIAPOL; EVE Ernst Vetter GmbH) was used for polishing of ceramic specimens in the following order: (I) blue, smoothing finisher; (II) pink, prepolisher; and (III) gray, high‐shine polisher. A low‐speed handpiece was used for this purpose, and each polisher was used for 1 min.

All ceramic specimens then underwent baseline assessment of color, surface roughness, and hardness, and the results were recorded quantitatively.

### 2.4 Baseline Color Assessment

The A3 shade of all ceramic types was used in this study for the purpose of standardization. Color assessment was performed with a spectrophotometer (VITA EasyShade Advance; VITA Zahnfabrik, Bad Säckingen, Germany) using the CIE L*a*b* color space. In this system, L* indicates lightness, a* indicates redness‐greenness, and b* indicates blueness‐yellowness.

The color of specimens was analyzed against a gray background three times, and the mean of the three values was calculated and recorded. The color change (Δ*E*) was calculated using the following formula:

ΔE=[Δa*2+Δb*2+ΔL*2]1/2.

### 2.5 Surface Roughness (Ra)

All ceramic specimens underwent baseline assessment of surface roughness by using a profilometer (Nemo Fanavaran Pars, Mashad, Iran), and the results were reported quantitatively in micrometers (μm).

### 2.6 Surface Hardness

All ceramic specimens also underwent a hardness test in a Vickers hardness tester (MH1 model, standardized with ASTM, NIST, and DIN; Kupa Pajuhesh Corporation, Sari, Iran). A 500 g force was applied on the surface of specimens for 10 s before bleaching, and each specimen was tested at three points. The mean of the three values was calculated and reported as the mean hardness number of the respective specimen. The results were reported in kgf/mm^2^.

### 2.7 Bleaching Procedure

Half of the ceramic specimens in each subgroup were subjected to bleaching with 15% CP (Opalescence PF 15%; Ultradent Product Inc., South Jordan, UT, USA) for 4 h/day while the other half underwent bleaching with 20% CP (Opalescence PF 20%; Ultradent Product Inc., South Jordan, UT, USA) for 3 h/day as per the manufacturer's instruction. The bleaching agent was applied on the prepared ceramic surface with a clean microbrush. The specimens were cleaned under running water after each bleaching step. The bleaching treatment was continued for 14 days, and then the specimens were stored in distilled water at 37°C.

### 2.8 Final Testing

Ceramic specimens underwent final assessment of color, surface roughness, and surface hardness after completion of bleaching treatment.

### 2.9 Statistical Analysis

Data were analyzed by SPSS version 24 (SPSS Inc., Armonk, IL, New York, USA). The changes in surface hardness and surface roughness of different ceramic types based on the type of surface treatment and concentration of CP were analyzed by repeated‐measures ANOVA, one‐way ANOVA, and post hoc Bonferroni test. Based on the Kolmogorov–Smirnov test, the data were checked for normality and the normality of the data was confirmed.

## 3 Results

### 3.1 Surface Roughness

Table 1 presents the mean Ra (μm) of the groups. As shown, the surface roughness (Ra) significantly increased in all groups after bleaching treatment. Comparison of surface roughness values before the bleaching treatment (to assess the effect of surface treatment on surface roughness) revealed a significant difference in surface roughness between polished and glazed specimens of Vita Mark II and Celtra Duo (*p* < 0.05), such that the glazed group had a significantly lower surface roughness than the polished group before the bleaching treatment. However, the difference in surface roughness between polished and glazed groups of Suprinity ceramic was not significant (*p* > 0.05). Regarding the effect of CP concentration, the results revealed a significant difference in surface roughness of polished Vita Mark II specimens exposed to 15% and 20% CP (*p* < 0.05) such that the group subjected to 20% CP showed higher surface roughness than the group exposed to 15% CP. However, the concentration of CP had no significant effect on surface roughness of other groups.

**Table 1:** Mean Ra (μm) of the groups.

Ceramic type	Surface treatment	CP concentration (%)	Before bleaching (mean ± SD)	After bleaching (mean ± SD)
Vita Mark ll	Polishing	15	250.16 ± 7.18^Aa^	300.97 ± 7.13^Ba^
		20	249.96 ± 7.51^Aa^	409.81 ± 7.91^Bb^
	Glazing	15	42.62 ± 2.23^Ab^	58.81 ± 2.88^Bce^
		20	43.02 ± 3.15^Ab^	75.17 ± 4.23^Bc^
Celtra Duo	Polishing	15	61.01 ± 19.75^Ac^	63.43 ± 20.77^Bc^
		20	60.87 ± 13.96^Ac^	62.72 ± 13.43^Bc^
	Glazing	15	30.49 ± 9.42^Ad^	32.43 ± 9.57^Bd^
		20	30.76 ± 3.68^Ad^	35.42 ± 5.05^Bd^
Suprinity	Polishing	15	34.28 ± 28.20^Abd^	40.79 ± 24.87^Bde^
		20	35.17 ± 6.74^Abd^	40.09 ± 8.82^Bd^
	Glazing	15	32.49 ± 8.09^Abd^	34.45 ± 8.52^Bd^
		20	32.75 ± 5.11^Abd^	35.23 ± 5.43^Bd^

*Note:* Similar uppercase letters indicate the absence of a significant difference in a row, while similar lowercase letters indicate the absence of a significant difference in a column at the 5% level of significance. Pairwise comparisons were made based on the post hoc Bonferroni test.

Abbreviation: SD, standard deviation.

### 3.2 Hardness

Table 2 presents the surface hardness of the groups. As shown, the surface hardness decreased in all groups after bleaching treatment but this reduction was only significant in Vita Mark II subgroups (polished and glazed, subjected to 15% and 20% CP). Comparison of hardness values before the bleaching treatment (to assess the effect of surface treatment on hardness) showed that the difference between the hardness of polished and glazed specimens was not significant in any ceramic group (*p* > 0.05). Regarding the effect of CP concentration, the difference between 15% and 20% CP was not significant with respect to hardness in any group.

**Table 2:** Mean surface hardness of the groups (kgf/mm^2^).

Ceramic type	Surface treatment	CP concentration (%)	Before bleaching (mean ± SD)	After bleaching (mean ± SD)
Vita Mark ll	Polishing	15	556.30 ± 45.60^Aa^	532.90 ± 51.41^Ba^
		20	561.10 ± 24.15^Aa^	548.50 ± 33.38^Bad^
	Glazing	15	576.60 ± 56.66^Aabc^	558.50 ± 41.28^Bacd^
		20	573.80 ± 18.29^Aabc^	536.20 ± 21.25^Bad^
CeltraDuO	Polishing	15	606.90 ± 35.76^Acd^	598.40 ± 33.43^Aab^
		20	604.20 ± 38.31^Acd^	595.40 ± 38.41^Aab^
	Glazing	15	640.40 ± 56.64^Ad^	637.50 ± 57.08^Ab^
		20	636.90 ± 62.38^Ad^	632.40 ± 62.92^Abc^
Suprinity	Polishing	15	619.30 ± 37.89^Acd^	612.00 ± 37.95^Abd^
		20	618.00 ± 67.95^Acd^	610.40 ± 68.91^Abd^
	Glazing	15	617.20 ± 60.61^Acd^	609.50 ± 59.58^Abd^
		20	617.30 ± 46.83^Acd^	612.40 ± 62.40^Abd^

*Note:* Similar uppercase letters indicate the absence of a significant difference in a row, while similar lowercase letters indicate the absence of a significant difference in a column at the 5% level of significance. Pairwise comparisons were made based on post hoc Bonferroni test.

Abbreviation: SD, standard deviation.

### 3.3 Color Change (Δ*E*)

**Figure 2:**
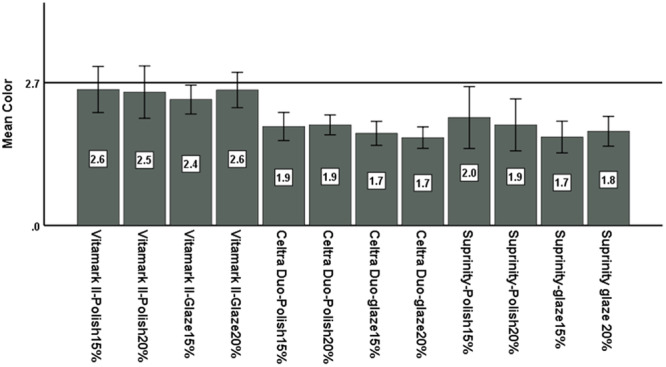
Mean Δ*E* of the groups.

Assessment of Δ*E* of different groups revealed the highest Δ*E* in polished Vita Mark II subjected to 15% CP (mean = 2.57) and the lowest Δ*E* in glazed Celtra Duo subjected to 20% CP (mean = 1.66). Also, considering Δ*E* ≤ 2.7 as clinically acceptable color change, and since Δ*E* in all groups was lower than this threshold, Δ*E* was not clinically and visually detectable in any group (Figure 2).

## 4 Discussion

The results of this study rejected the null hypothesis about surface roughness CP treatment caused a significant increase in surface roughness in all studied ceramic groups. Regarding surface hardness, the null hypothesis was rejected in the vita mark II subgroups, and in the other subgroups, treatment with CP did not have a statistically significant effect on surface hardness. The null hypothesis about color changes in all ceramic groups was accepted.

This study assessed the effects of 15% and 20% CP on color, surface roughness, and hardness of glazed and polished CAD/CAM dental ceramics. CP was used in the present study since it is the most commonly used bleaching agent for home bleaching (Leite et al. 2023). Following application, CP releases hydrogen peroxide and free radicals that whiten the tooth (Meireles et al. 2008). In this process, H+ and H_3_O+ released from CP enable selective passage of alkaline ions and result in degradation of the glass network of ceramic, leading to degradation of dental porcelain, and the resultant change in its surface properties (Demir, Karci, and Ozcan 2020). In home bleaching, teeth are exposed to the bleaching agent for 2–8 h/day, depending on the manufacturer's instructions (Rea et al. 2019). Thus, in this study, dental ceramics were exposed to CP for 3–4 h/day for 14 days.

The results of this study showed that the surface roughness (Ra) significantly increased in all groups after bleaching treatment. The surface hardness decreased in all groups after bleaching treatment but this reduction was only significant in Vita Mark II subgroups. Assessment of Δ*E* of different groups revealed the highest Δ*E* in polished Vita Mark II subjected to 15% CP (mean = 2.57) and the lowest Δ*E* in glazed Celtra Duo subjected to 20% CP (mean = 1.66). Δ*E* was not clinically and visually detectable in any group.

Studies on the effects of bleaching agents on surface properties of ceramics are limited (Çağrı et al. 2014). Zaki and Fahmy (2009) showed that surface roughness above the threshold of Ra= 0.2 µm can enhance plaque accumulation and increase the risk of secondary caries and gingivitis. It can also change the ceramic surface and adversely affect its esthetic appearance. Butler et al. (2004) reported a significant increase in surface roughness of ceramics exposed to 10% CP for 21 days. Similar results were obtained in the present study using 15% and 20% CP. Duschner et al. (2004) found no significant change in surface morphology of porcelain ceramics exposed to dental bleaching agents, which was probably due to the use of low‐concentration bleaching agents. Zaki and Fahmy (2009) showed that bleaching significantly increased the surface roughness of polished over‐glazed ceramics. This result was in agreement with the findings of White et al. (2003), Rosentritt et al. (2005), and Silva et al. (2006). The increase in surface roughness can be due to etching of ceramic surface with CP, although Zavanelli et al. (2011) reported no significant change in surface properties of ceramics exposed to 10% and 15% CP for 126 h. In Türker and Biskin (2002) in their spectral analysis of ceramic surfaces revealed a reduction in SiO_2_, and K_2_O_2_ contents of feldspathic porcelain by 4.82% and 1.89%, respectively, compared with their original content following exposure to CP (Türker and Biskin 2002). In the present study, comparison of surface roughness values before the bleaching treatment (to assess the effect of surface treatment on surface roughness) revealed a significant difference in surface roughness between polished and glazed specimens of Vita Mark II and Celtra Duo, such that the glazed group had a significantly lower surface roughness than the polished group before bleaching treatment. However, the difference in surface roughness between polished and glazed groups of Suprinity ceramic was not significant.

Glazing increases the fracture resistance of ceramics and decreases their wear potential by filling the porosities on the surface of heated porcelain (Demir, Karci, and Ozcan 2020). Moreover, glazing decreases the surface roughness by reduction of surface porosities, and resultantly, minimizes the colonization of microorganisms such as *Candida albicans* on ceramic surfaces in the oral cavity (Demir, Karci, and Ozcan 2020). This statement can show better surface properties of glazed ceramics in the present study, although the polished and glazed surfaces of Suprinity specimens had no significant mechanical difference with each other, and thus, both showed almost similar surface roughness values. The same results were reported by Kurt et al. (2020).

Regarding the effect of concentration of CP, the present results showed that after bleaching treatment, a significant difference was noted only between the effects of 15% and 20% CP on surface roughness of polished Vita Mark II specimens, such that the group subjected to 20% CP showed higher surface roughness than the group exposed to 15% CP. However, concentration of CP had no significant effect on surface roughness of other groups. This finding can be due to the effect of higher concentrations of CP on the glass matrix of ceramics. The glass matrix of feldspathic ceramics is degraded following exposure to higher concentrations of CP, and resultantly, the surface roughness increases. This result was in agreement with the results of Kwon and Wetrz (2015), who showed that higher concentration of CP had a greater effect on surface properties of dental restorative materials.

No significant increase in surface roughness of Suprinity and Celtra Duo ceramics following exposure to CP can be due to their higher chemical stability. Suprinity and Celtra Duo are silica‐based zirconia‐reinforced glass ceramics with a mean crystal size of 0.5 µm. Their excellent fine structure results in their smooth surface and their high zirconium dioxide content confers resistance against surface roughness changes (Vichi et al. 2018).

Hardness of a material is related to its stiffness, proportion limit, and wear potential by the opposing teeth (Demir, Karci, and Ozcan 2020). Thus, any chemical softening due to bleaching can affect the durability of restorative materials. In the present study, the surface hardness of all groups decreased after bleaching treatment; however, this reduction was only significant in Vita Mark II subgroups (polished, glazed, 15% and 20% CP). Comparison of values before bleaching (to assess the effect of surface treatment on surface hardness) revealed no significant difference between polished and glazed specimens in any ceramic group. Also, regarding the effect of concentration of CP, the results showed no significant difference in hardness between 15% and 20% CP in any ceramic group. A previous study reported a significant reduction (15%) in microhardness of self‐glazed feldspathic ceramics after bleaching with 10% and 16% CP for 8 h/day for 30 days (Alshali and Alqahtani 2022). This reduction in microhardness can be due to a reduction in SiO_2_ content, which is the main constituent of the glass matrix of all glass–ceramic materials, as shown by spectral analysis of the surface of feldspathic porcelain after bleaching treatment (Alshali and Alqahtani 2022). A reduction in silicon, potassium, and aluminum contents has been reported by elemental analyses of different ceramics immersed in acidic drinks, which indicates that ceramics are sensitive to low pH solutions and oxidizing agents that cause their chemical degradation (Alshali and Alqahtani 2022). The hardness of Celtra Duo and Suprinity ceramic specimens did not significantly change following exposure to bleaching agents in the present study, which highlights their higher chemical stability as the result of their higher crystalline content of tetragonal zirconia, compared with other ceramic types (Zarone et al. 2021).

In a study by Alshali and Alqahtani (2022), the surface hardness of Celtra Duo ceramic specimens did not experience a significant change after bleaching. Malkondu et al. (2011) revealed that CP significantly decreased the microhardness of leucite‐reinforced glass ceramics, conventional glass ceramics, and feldspathic porcelains. Also, Türker and Biskin (2002) showed a reduction in microhardness of feldspathic porcelain after bleaching with CP. However, Zavanelli et al. (2011) and Polydorou et al. (2007) demonstrated that the surface hardness of ceramics was not affected by bleaching treatment. Controversy in the reported results in this respect can be due to differences in exposure times to bleaching agents, and using different types and concentrations of bleaching agents.

Assessment of color change of ceramics after bleaching in the present study revealed the highest Δ*E* in polished Vita Mark II exposed to 15% CP (mean = 2.57) and the lowest Δ*E* in glazed Celtra Duo subjected to 20% CP (mean = 1.66). Considering Δ*E* ≤ 2.7 as a clinically acceptable color change, Δ*E* was not clinically and visually detectable in any group. Although Δ*E* values were reported as the highest and lowest, the highest Δ*E* belonged to Vita Mark II subgroups with the smallest difference from the threshold of 2.7. The Δ*E* values of Celtra Duo and Suprinity were highly close and had the greatest difference from the threshold of 2.7. Such results were expected considering the excellent chemical stability and mechanical properties of these ceramics. Alshali and Alqahtani (2022) demonstrated that the Δ*E* of Celtra Duo after bleaching was within the clinically acceptable range. Rodrigues et al. (2019) revealed significant color change of feldspathic ceramics following exposure to bleaching agents. The highest Δ*E* in Vita Mark II and the lowest Δ*E* in Celtra Duo in the present study cannot be directly attributed to changes in hardness because hardness and color are two independent surface properties (mechanical vs. optical) that are affected by different parameters. Nonetheless, this finding may be due to changes in surface roughness after bleaching that can affect color by surface alterations (Ghinea et al. 2011). The present results showed a direct correlation between color change and surface roughness such that the highest Δ*E* and the highest increase in surface roughness were both noted in polished Vita Mark II ceramic specimens, and the lowest Δ*E* and the smallest increase in surface roughness were observed in glazed Celtra Duo ceramic. This finding was in agreement with the results of Tinastepe et al. (2021).

Considering all the above, dental clinicians should be careful in conduction of bleaching treatment for patients with ceramic restorations, and should preferably use barriers to protect the ceramic restorations. In case of accidental exposure to bleaching agent, polishing of ceramic may be required to minimize the adverse effects of bleaching agent.

This study had an in vitro design. Saliva and masticatory forces which are important parameters that can affect the mechanical response of restorative materials before and after bleaching were absent in the present study. Thus, generalization of results to the clinical setting must be done with caution. Future in vivo studies are recommended to obtain more reliable results. Furthermore, future studies should assess the effects of longer exposures to bleaching agents on surface properties and color of ceramics to find out whether such changes are time‐dependent.

## 5 Conclusion

The present results revealed that concentration of CP and type of surface treatment affected the surface properties of CAD/CAM ceramics. Type of surface treatment only affected the surface hardness of Vita Mark II ceramics. Concentration of CP had a significant effect only on polished Vita Mark II.

## Author Contributions

Study concept and design: Homayoun Alaghehmand and Saman Jamshidi. Acquisition of data: Saman Jamshidi. Analysis and interpretation of data: Homayoun Alaghehmand and Saman Jamshidi. Drafting of the manuscript: Saman Jamshidi. Critical revision of the manuscript for important intellectual content: Homyoun Alaghehmand. Statistical analysis: Hemmat Gholinia. Administrative, technical, and material support: Homayoun Alaghemand. Study supervision: Homayoun Alaghehmand and Behnaz Esmaeili.

## Conflicts of Interest

The authors declare no conflicts of interest.

## Data Availability

The authors have nothing to report.
